# A new semislug of the genus *Laocaia* (Gastropoda, Pulmonata, Helicarionidae) from Vietnam

**DOI:** 10.3897/zookeys.846.34372

**Published:** 2019-05-16

**Authors:** Ivaylo Dedov, Ulrich Schneppat, Manh Quang Vu, Nguyen Quoc Huy

**Affiliations:** 1 Institute of Biodiversity and Ecosystem Research, Bulgarian Academy of Sciences, 2 Gagarin Street, 1113 Sofia, Bulgaria Institute of Biodiversity and Ecosystem Research, Bulgarian Academy of Sciences Sofia Bulgaria; 2 CH-7074 Churwalden-Malix, Sennereiweg 8, Switzerland Unaffiliated Churwalden-Malix Switzerland; 3 Ho Chi Minh City University of Food Industry, 140 Le Trong Tan St., Tan Phu, Ho Chi Minh City; c/o Hanoi National University of Education (HNUE), 136 Xuan Thuy Rd., DHSP Cau Giay, Hanoi, Vietnam Ho Chi Minh City University of Food Industry Ho Chi Minh Vietnam; 4 Institute of Ecology and Works protection, 267 Rd. Chua Boc, Dong Đa, Hanoi, Vietnam Institute of Ecology and Works protection Hanoi Vietnam

**Keywords:** Helicarionidae, *
Laocaia
*, new species, Vietnam

## Abstract

A new species of the genus *Laocaia* Kuzminykh, 1999, *Laocaiasimovi* Dedov & Schneppat, **sp. nov.**, is described, which was collected from a single locality in northern Vietnam. Color pictures of living specimens are provided. For the first time, information on the ecology and biology of a representative of the genus *Laocaia* is presented.

## Introduction

The genus *Laocaia* was described by Kuzminykh (1999) and contains two species – *Laocaiaattenuata* Kuzminykh, 1999 and *L.obesa* Kuzminykh, 1999 – from the area of the Fansipan Mountain massive, northern Vietnam. Both species were recorded from relatively high altitudes between 1800–2000 m in the forest zone in the vicinity of Fansipan. The habitats for both species were not explicitly described by Kuzminykh (1999), and the type localities were not georeferenced. Until recently, no additional information on other species or the biology of members of the genus were published (Schileyko 2011).

Our finding of a third species strongly suggests that the genus has evolved in the ecological niches of the high altitudes of the Fansipan Mountain tops, and records of further new species of the genus can be expected in the future. The potential type locality of *L.attenuata* is situated only 3.5 km eastwards, and that of *L.obesa* can be found about 4.8 km in a southeast direction of the type locality of *Laocaiasimovi* Dedov & Schneppat, sp. nov., on the same mountain massive. This indicates a high degree of endemism in a comparatively small area, which is in urgent need of further research. According to Páll-Gergely et al. (2015), the molluscan fauna of the border region of Sơn La and Yen Bái Provinces (Phan Xi Păng = “Fansipan” Mountain and its vicinity) is nearly unknown. This may be due to the high abundance of limestone-free bedrock causing a relatively depleted richness and diversity of land molluscs.

## Material and methods

**Figure 1.**
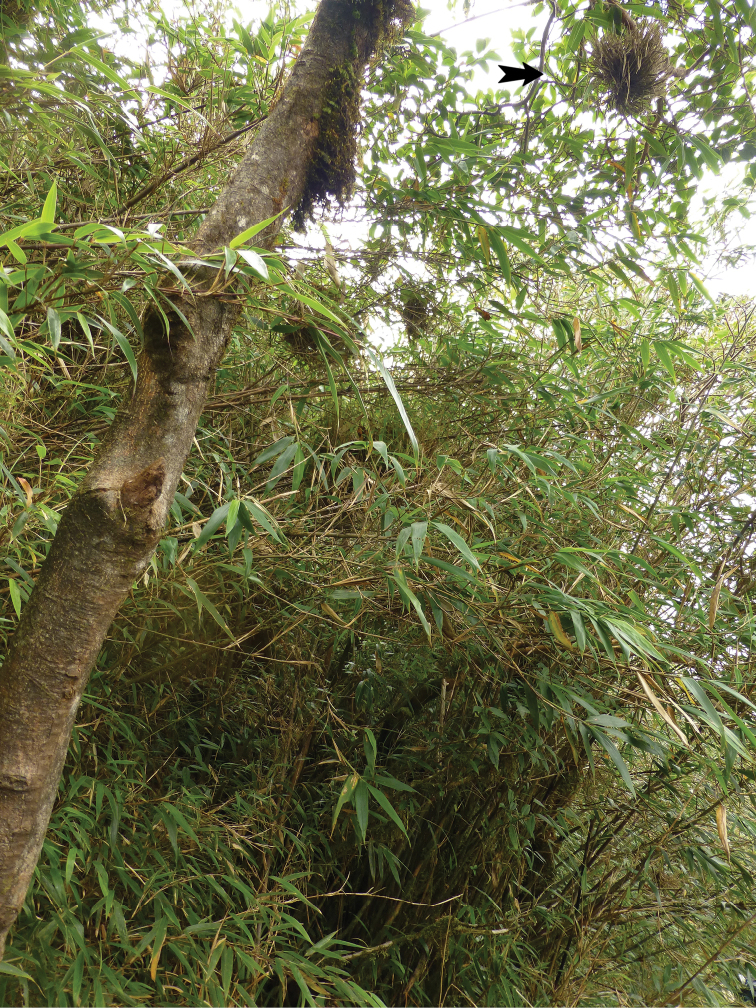
*Laocaiasimovi* Dedov & Schneppat, sp. nov. habitat picture, from the type locality near the peak of Fansipan. The black arrow points towards the epiphytic plants, in which the new species was found.

Abbreviations: **IBER-BAN** – Institute of Biodiversity and Ecosystem Research, Bulgarian Academy of Sciences; **NMNHS** – National Museum of Natural History, Sofia, Bulgaria.

The specimens were collected with an entomological standard sweep net from elevated branches of a leaf bearing tree of unknown species in the forest, on the eastern slope of the top of Fansipan Mountain (Fig. 1).

The treatment of the specimens followed Nitz et al. (2009: 280), directly after collecting in the evening of the same day. One specimen was photographically documented alive prior to killing and preservation, using a digital camera Panasonic, Lumix DMC-TZ31. Because of the delicateness of the preserved specimens these were soaked for some minutes in a 1% NaCl-solution to relax them and to prevent desiccation and malformation during the measuring process under the stereo microscope. All measurements have been taken from preserved animals. The measurements were taken under a stereo-microscope with a scaled ocular and transformed into millimeters.

### Morphological studies

All measurements given are from specimens preserved in ethanol 75%. The total length and body width, mantle length and mantle width, length of free mantle flap, sole length and sole width, height and width of genital pore of the three adults of the series were measured. Maturity was determined prior to dissection by examining the genital pore under a binocular microscope. The general method of dissecting follows Wiktor (2000: 383–384).

## Results

### Family Helicarionidae Bourguignat, 1877

#### Subfamily Helicarioninae Bourguignat, 1877

##### Genus Laocaia

Taxon classificationAnimaliaStylommatophoraHelicarionidae

Kuzminykh, 1999

###### Type species.

*Laocaiaattenuata* Kuzminykh, 1999.

###### Diagnosis.

small slugs with non-coiled visceral hump, rounded posteriorly, lying in V- shaped body groove; body cavity not extending into tail; shell very thin, non-spiral, internal, hemispheric, completely covering visceral hump, with small calcified part; penis short, bulbous, with large stimulator inside; epiphallus and flagellum absent; spermatheca entering atrium between vagina and penis (amended after Kuzminykh 1999).

###### Remarks.

for the systematic position of the genus *Laocaia*, we here follow MolluscaBase (2018). The genus was originally placed in the family Ariophantidae by Kuzminykh (1999), which was followed by Bouchet et al. (2017). However, Schileyko (2002, 2011) transferred it to the family Helicarionidae.

##### Laocaia simovi

Taxon classificationAnimaliaStylommatophoraHelicarionidae

Dedov & Schneppat sp. nov.

http://zoobank.org/5106D544-8BDC-4BBF-B04D-EEB763616827

**Figure 2.**
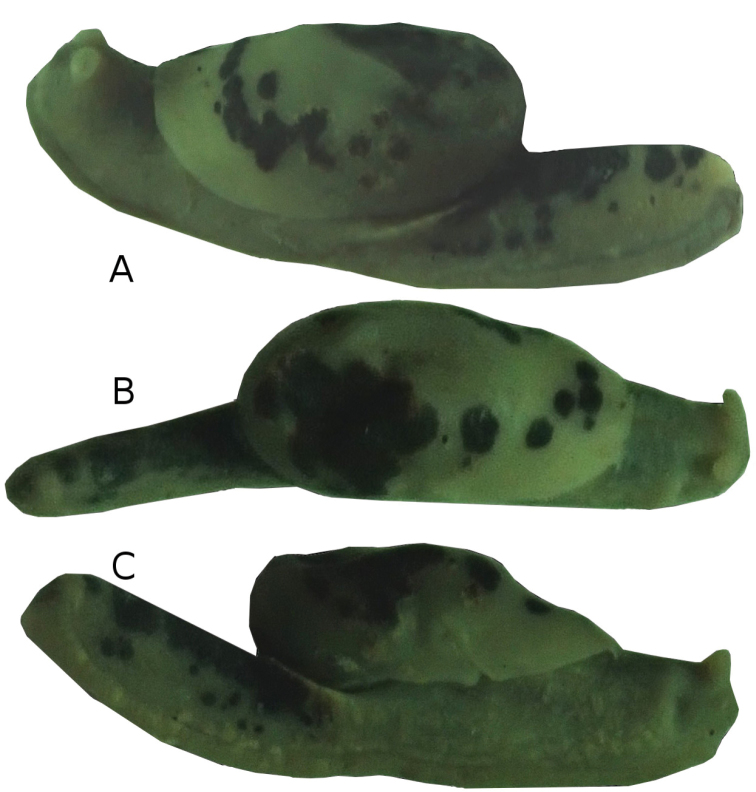
*Laocaiasimovi* Dedov & Schneppat, sp. nov. holotype specimen in lateral left (**A**), dorsal (**B**), and lateral right (**C**) views.

**Figure 3.**
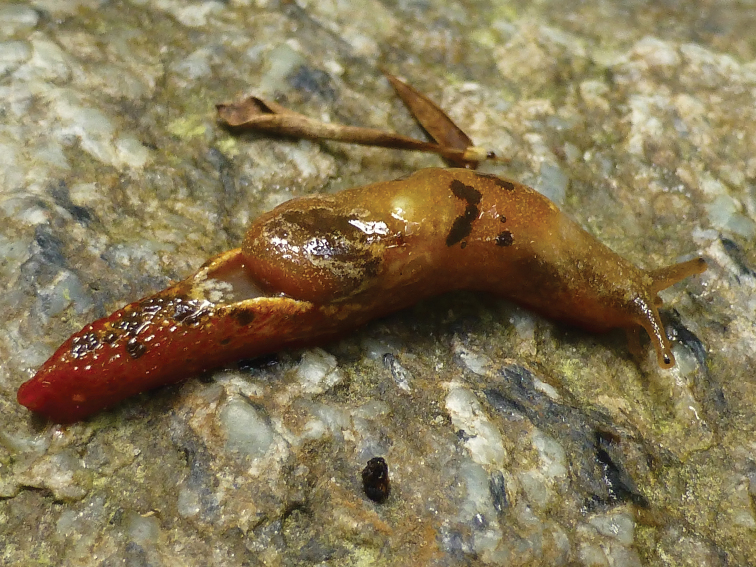
*Laocaiasimovi* Dedov & Schneppat, sp. nov. coloration of the body with the visceral hump and U-shaped dorsal groove.

**Figure 4.**
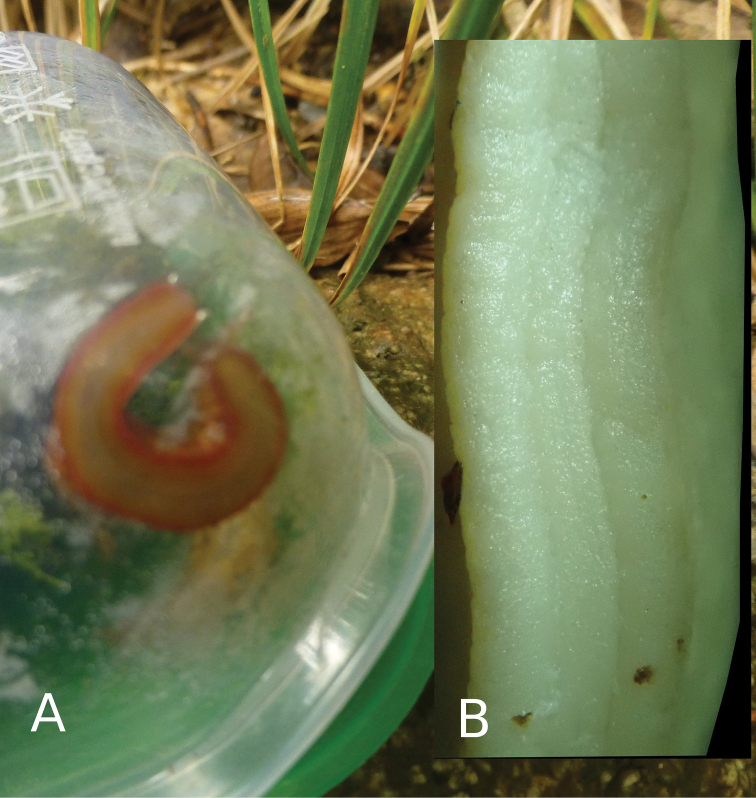
*Laocaiasimovi* Dedov & Schneppat, sp. nov. sole coloration and sole zones in a living specimen (**A**) picture was taken in situ in the field, when the first animal was collected in the box, and sole of preserved animal (**B**).

**Figure 5.**
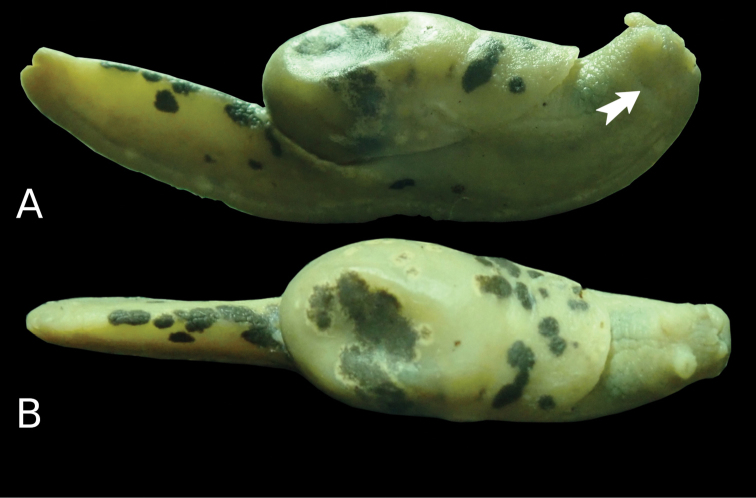
*Laocaiasimovi* Dedov & Schneppat, sp. nov. preserved specimen, paratype IBER-BAN 40339/1-C, lateral right (**A**) and dorsal (**B**) view. The white arrow points to the genital pore.

**Figure 6.**
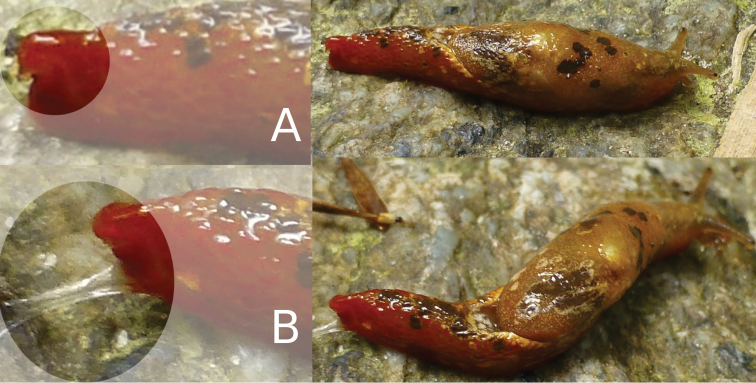
*Laocaiasimovi* Dedov & Schneppat, sp. nov. pointed posterior end of the dorsum with the horn-like structure (**A**) and the coloration of the slime (**B**).

**Figure 7.**
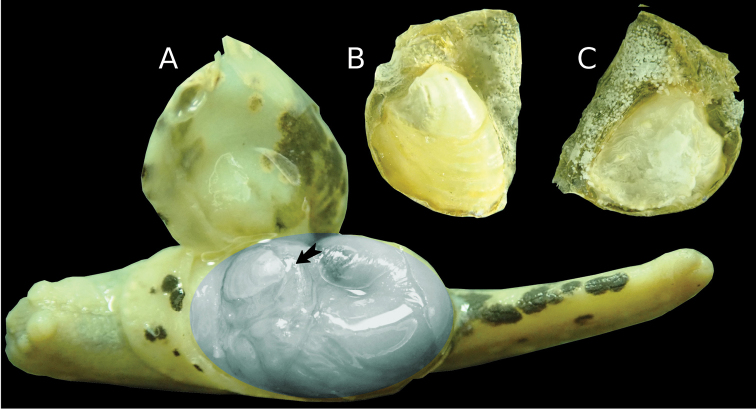
*Laocaiasimovi* Dedov & Schneppat, sp. nov. vestigial shell (**A**) position of its calcified part on anterior roof of the visceral hump indicated by black arrow, the visceral hump indicated by grayish ellipse; dorsal view of the shell (**B**) ventral view of the shell (**C**).

**Figure 8.**
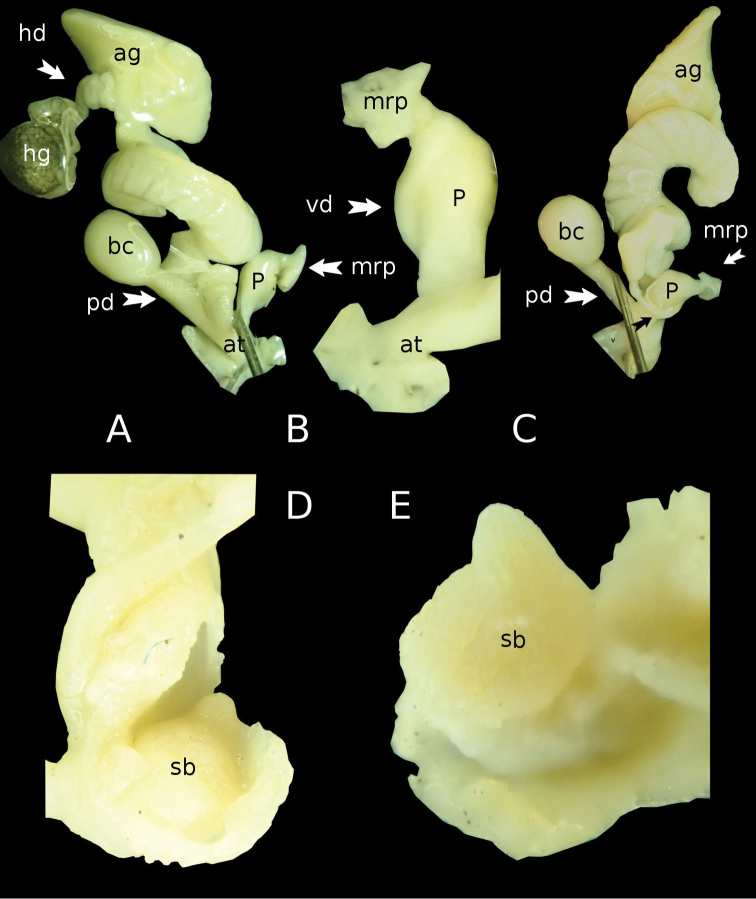
*Laocaiasimovi* Dedov & Schneppat, sp. nov. genital anatomy, the whole sexual system (**A**), male part of the sexual system (**B**), sexual system, opposite view (**C**), position of the sarcobelum inside of the penis (**D**), and inverted (**E**). Abbreviations: **p** – penis; **sb** – sarcobelum, **mrp** – musculus retractor penis, **vd** – vas deferens, **at** – atrium, **bc** – bursa copulatrix, **pd** – pedunculus, **ag** – albumen gland, **hg** – hermaphroditic gland, **hd** – hermaphroditic duct.

Figs 2 , 3 , 4 , 5 , 6 , 7 , 8

###### Holotype.

VIETNAM: Lao Cai Province, Fansipan Mountain below peak, in monsoon influenced leaf-bearing mountain forest with dense undergrowth of bamboo, ca 2990 m; 22.30560°N, 103.77625°E; 21 Sep 2018; I. Dedov, N. Simov, R. Bekchiev, P. Beron leg.; NMNHS 10805, ex. coll. IBER-BAN 40339/1-A.

###### Paratypes.

2 adults and 2 juveniles, same data as for holotype: NMNHS 10806, ex. coll. IBER-BAN 40339/1-E; coll. IBER-BAN, 40339/1: B, C, D.

The paratype IBER-BAN 40339/1-C is shown in the photos of the living semislug, as well as in all photos of the preserved animal and its anatomy.

###### Measurements of holotype.

Holotype: total length 24.12 mm, body width 6.72 mm, mantle length 15.96 mm, mantle width 6.72 mm, free mantle flap length 4.08 mm, sole length 24 mm, sole width 3.36 mm, tale length 8.28, tale width 2.52 mm, tale “horn” length 1.3 mm, tale “horn” width 1 mm, genital pore length 1.05 mm, genital pore width 0.25 mm (Fig. 2).

###### Differential diagnosis.

Externally, *L.simovi* Dedov & Schneppat, sp. nov. differs from *L.attenuata* and *L.obesa* by its coloration. The anterior body of *L.attenuata* is yellow, and its head and neck show three indistinct stripes, while the mantle is marbled with irregularly arranged black spots. The posterior part of the foot is uniformly grayish. In contrast, the body of *L.obesa* is whitish-grayish colored and covered with numerous white spots. On the visceral hump, the mantle of this species displays a pattern of irregularly arranged black spots (Kuzminykh 1999).

Anatomically, *L.simovi* sp. nov. differs from *L.attenuata* by the narrower base of the penis, as well as by its much more globular sarcobelum with a pointed, cone-shaped apical tip. The sarcobelum is not covered with numerous distinct papillae, but it shows very fine longitudinal striae. The striation is not well visible even under high magnification.

*Laocaiasimovi* sp. nov. differs from *L.obesa* by its less voluminous penis and the narrower base of the penis. The inner wall of the penis is not covered with papillae, but with almost invisible, very fine granules. The bursa copulatrix of *L.simovi* sp. nov. is of almost globular shape, the pedunculus is long, slender and of the same diameter all along its length; its bursa copulatrix is more voluminous than the penis.

###### Description.

*Coloration*: The primary color of the anterior body is light-ocher-brownish. The head, including the ommatophores, is of a grayish-ocher color. The black eye spot on top of the ommatophores is contrasting and well visible. The “tale”, e.g. the posterior part of the foot is of a deep brick red color. This coloration continues anteriorly until the lower sides of the head, where it fades. The whole body shows a pattern of irregularly dispersed, white-yellowish and brownish-blackish spots and markings, which are also irregular in size, shape and placement between specimens. The brownish-blackish spots are missing on the neck and lower frontal sides of the body. The well pronounced dorsal edge of the “U” shaped dorsal groove behind the visceral hump is yellowish (Fig. 3). The sole in living specimens is subdivided in two reddish lateral sole fields and a central creamy-yellowish field (Fig. 4A). In preserved animals, the complete sole loses its differentiating pigmentation (Fig. 4B), which is also the case for all other body-parts of the specimens. All colorful pigments (besides melanin) are dissolved in ethanol during the preservation process (Fig. 5A, B). On the entire body, the slime is thin, transparent and colorless (see Fig. 6B). There was no defensive slime of different color or consistence observed in living specimens.

*Body*: The body (n = 3, adult specimens) is slender, elongated, and of comparatively small size and fits the known body dimensions of species within the genus *Laocaia*. The total length of preserved adult specimens is 21.12–26.40 mm. The body width is 5.76–6.72 mm. The visceral hump length is 11.52–15.96 mm. The visceral hump width is equal to the body width. The free mantle flap is 3.60–4.08 mm. In living animals, the integument of the anterior part of the body, including the mantle over the visceral hump, is very finely granulated, but looks smooth on the photo of a living specimen (Fig. 3). However, the integument of the posterior part of the foot, as well as of the body flanks, is visibly roughly sculptured with big and well-rounded granules, which appear almost like the wrinkle rows in other slugs. This sculpture of the integument disappeared in all specimens during preservation, and not even the grooves between the sculptural elements remained (Fig. 5A, B). The posterior part of the foot is laterally depressed and formed like a steep roof. Observed with the naked eye, it resembles a keel structure, but there is no real keel like in Limacidae, for example. The length of the posterior part of the foot is 7.08–9.48 mm; the width is 2.40–2.64 mm. The dorsum has a little hornlike prolongation at its posterior end. The posterior horn length is 1.30–1.32 mm; the posterior prolongation width is 0.72–1.00 mm. The posterior prolongation is slightly longer than the foot in living animals (see Fig. 6A). The posterior prolongation is separated from the dorsum with a slit and lays on it (observed in preserved specimens). The enlarged and bubble-shaped visceral hump with the entity of the visceral mass is lying in the body cavity. The mantle fully covers the shell, also in the juvenile specimens, and no slit was observed. The body cavity is not enlarged into the posterior part of the foot, which is fully muscular. The sole is narrow, only about a third of the body width, divided in three longitudinally fields, equally finely sculptured resembling small blisters (see Fig. 4B). The sole length is 21.26–26.04 mm, sole width 3.00–3.36 mm, the lateral zones 1.08–1.2 mm, and the central zone 0.84–0.96 mm. The pneumostome is located more or less centrally of the right of the mantle. The wide and deep dorsal U-shaped groove behind the visceral hump, as well as the lack of organs, except muscles in the posterior part of the foot, allows *Laocaiasimovi* sp. nov. to move its raised “tale” very fast from side to side (refer to biological and ecological observations). In mature animals, the genital pore is well visible, about 2.1–2.6 mm posterior to the front on the right side of the neck, a little below the base of the right ommatophore. The length of the genital pore is 1.05–1.10 mm; the width is 0.25–0.30 mm (Fig. 5A).

*Shell*: The vestigial shell is placed on the anterior roof of the visceral hump. The more calcified apical part of it is situated on the anterior part of the shell. The whole shell is found somewhat central right from the body axis. In other known vestigial shells, the small calcified part is situated apically. The thin seam of periostracum is posterior to the hard plate, rather than anterior to it in other *Laocaia* species. The apex is well distinguished and positioned almost centrally. The whole vestigial shell is completely covered with the mantle. It is difficult to extract it out of the shell-bed without damage. The shell is very thin and transparent. Under high magnification it is grainy, calcareous from ventral, except the much harder, triangulate, more massive calcified plate of creamy-white opaque color. The shell is without any remains of a helicoid apex, more like those, usually found in Limacidae, Agriolimacidae or Milacidae (Fig. 7A–C). The periostracum is somewhat glossy and of pale-yellow color.

*Anatomy of genital organs* (Fig. 8A–E): ♂-parts: the penis (p) is relatively short, widened in its middle section and generally fusiform, narrow in its basal part – length: 2.80 mm, width – base: 1.08 mm, – middle section: 1.56 mm. The walls of the penis are muscular, its interior walls are very finely granulated. Inside the penis, at its posterior end, a globular sarcobelum (sb), with a pointed, cone-shaped apical tip. Its surface is fine longitudinally striated, but the striation is not well visible even under high magnification (Fig. 7D, E). Below the sarcobelum, there is the simple opening of the vas deferens. The musculus retractor penis (mrp) is short and thick. Its length is 0.84 mm, width is 0.33–0.66 mm. The musculus retractor penis is attached almost apically to the posterior end of the penis. The vas deferens (vd) is short and thinner than the penis. It attaches to the outer wall of the penis and inserts at the penis aside the mrp. The vas deferens is wrapped tightly with connective tissue around the penis (Fig. 8B). An epiphallus or a flagellum is absent.

♀-parts: Bursa copulatrix (bc) is big, almost spherical – length: 3.09 mm, width: 2.40 mm, with a relatively long pedunculus (pd) – length: 4.62 mm, width: 0.93 mm over its complete length. A spermatophore was not found in the pedunculus or bursa copulatrix. The albumen gland (ag) is very large and pale yellowish-white – length: 4.14 mm, width: 2.55–2.76 mm. The free oviduct (fod) is basally conical and then twisted and folded with approximate length of 3.7 mm and a width of 0.9–1.1 mm. It inserts to the atrium at almost the same position as the penis.

♂♀-parts: The atrium (at) is short and tubular. The hermaphroditic gland (hg) is big, relatively globular with a length of 3.6 mm and a width of 2.4 mm, gray-brownish and covered with a dense blackish, irregular connective tissue. The hermaphroditic duct (hd) is short, twisted and folded all along its length with a width of 0.54–0.6 mm. The spermoviduct (sod) is of 1.11–1.14 mm width.

*Biological and ecological observations*: Up to now, the species was found in epiphytic plant-groups, on elevated branches, in the tree crowns in the forests just below the Fansipan peak. If the animal is touched, it first tries to escape very fast, but if caught, the semislug takes a position with the head inverted and starts to wag its raised “tail” very fast from one side to the other, probably imitating land leeches (Hirudinea). This behavior can be interpreted to be a protective measure against predators, but also to flip itself down from twigs or leaves to the forest floor in order to escape a potential predator. Similar observations of rapid “tail movement” are published by Wiktor (2002) for the semislug species *Cryptausteniasaltatoria* Wiktor, 2002 and *Cryptausteniaobesa* Wiktor, 2002 (Helicarionidae) in New Guinea.

###### Distribution.

Up to now this species is known only from its type locality.

###### Derivatio nominis.

This new species is named after our friend and biologist Dr. Nikolay Simov, NMNHS, Sofia, Bulgaria, who found the first specimen of the species.

## Supplementary Material

XML Treatment for Laocaia

XML Treatment for Laocaia simovi
